# Evaluation of Lymphadenopathy in Urgent Care: A Case of Hodgkin Lymphoma

**DOI:** 10.7759/cureus.115623

**Published:** 2026-09-01

**Authors:** Yeli Wang, Michael D Yashar

**Affiliations:** 1 Medicine, David Geffen School of Medicine at UCLA (University of California, Los Angeles), Los Angeles, USA

**Keywords:** axillary lymphadenopathy, cervical lymphadenopathy, clinical-pathologic discordance, false-negative results, hodgkin lymphoma, lymphoproliferative disorder, multistation lymphadenopathy, nodular sclerosis type, oncology, urgent care

## Abstract

Lymphadenopathy is a common presentation in urgent care and primary care settings and is most often due to benign or infectious causes. However, persistent or unexplained lymphadenopathy may represent an underlying malignancy, including Hodgkin lymphoma. We present the case of a 20-year-old female patient who presented to urgent care with a two-week history of cervical and axillary lymphadenopathy without B symptoms. Initial laboratory evaluation was notable for a positive mumps IgM, suggesting an infectious etiology. However, imaging revealed multistation cervical, axillary, and mediastinal lymphadenopathy concerning for a lymphoproliferative disorder. An initial core needle biopsy of an axillary lymph node was non-diagnostic; however, due to discordance between imaging and pathology findings, a repeat biopsy was performed, which confirmed classical Hodgkin lymphoma, nodular sclerosis subtype. The patient was subsequently referred for further oncologic evaluation and treatment. This case highlights the diagnostic challenges of evaluating lymphadenopathy when initial findings suggest an infectious process and emphasizes the importance of repeat biopsy when clinical suspicion for malignancy remains high.

## Introduction

Hodgkin lymphoma is the most common lymphoma in adolescents and young adults, with an incidence of approximately two to three cases per 100,000 persons per year in developed countries [1]. It demonstrates a bimodal age distribution, with peaks in adults between 20-30 years and 50-70 years of age. Classical Hodgkin lymphoma accounts for approximately 95% of cases and includes four histologic subtypes, with nodular sclerosis being the most common [2].

Risk factors for Hodgkin lymphoma include Epstein-Barr virus (EBV) infection, genetic susceptibility, immunodeficiency, and autoimmune disease [2]. Hodgkin lymphoma typically presents with painless cervical or supraclavicular lymphadenopathy and may be associated with mediastinal involvement and B symptoms, including fever, night sweats, and weight loss [3]. However, early presentations may be nonspecific and can mimic benign or infectious causes of lymphadenopathy, which may delay diagnosis.

Lymphadenopathy is a common presentation in urgent care and primary care settings, and the majority of cases are benign and self-limited [4]. The prevalence of malignancy among patients presenting with lymphadenopathy in primary care is estimated to be approximately 1.1%, compared with 20.8% in referred populations [5,6]. Therefore, recognizing findings that warrant further evaluation and reconciling discordant clinical, radiologic, and pathologic findings are important to avoid delayed diagnosis.

We present a case of classical Hodgkin lymphoma in a young patient who initially presented to urgent care without B symptoms and with an isolated positive mumps IgM, despite imaging findings concerning for a lymphoproliferative process. The initial core needle biopsy was nondiagnostic due to limited lymph node sampling, prompting repeat biopsy because of persistent clinical-radiologic concern. This case highlights the importance of maintaining suspicion for malignancy despite potentially reassuring or misleading initial findings and of pursuing further tissue sampling when biopsy results are discordant with the clinical and radiologic presentation.

## Case presentation

A 20-year-old female patient with no significant past medical history presented to urgent care with a two-week history of concern for left-sided cervical lymphadenopathy. The patient initially noticed palpable, tender bumps along the left side of her neck. One week prior to presentation, she developed swelling and pain in the left axillary region, rated 6/10 in severity, followed by intermittent left-sided pleuritic chest pain three days prior to presentation. She denied fever, night sweats, unintentional weight loss, sore throat, headache, nasal congestion, cough, abdominal pain, rash, extremity pain or swelling, focal weakness or numbness, speech changes, recent travel, recent surgery, or history of deep vein thrombosis, pulmonary embolism, or tuberculosis. She reported use of a levonorgestrel intrauterine device and endorsed no concern for pregnancy. She was up to date on her childhood immunizations. Her medical history was notable for a prior episode of cervical lymphadenopathy 18 months earlier, at which time testing for streptococcal pharyngitis and mononucleosis was negative. Recent outpatient testing for HIV and syphilis was also negative. Family history was notable for non-Hodgkin lymphoma in a great-aunt diagnosed in her 40s.

On physical examination, the patient was afebrile with normal vital signs, appeared well, and was in no acute distress. Examination of the neck revealed palpable, tender anterior and posterior firm cervical nodules on the left side without overlying erythema, warmth, or fluctuance. Breast examination demonstrated fullness in the left axillary region with mild tenderness to palpation but no overlying erythema, warmth, or fluctuance. No discrete palpable lymph nodes or masses were identified in the breast tissue, and there was no expressible nipple discharge. There was notable asymmetry between the left and right axillary regions. No additional lymphadenopathy was appreciated. The abdomen was soft and non-tender with no palpable masses. The remainder of the physical examination was unremarkable.

Due to the patient's acute and nonspecific presentation, an extensive diagnostic workup was initiated in urgent care. Laboratory evaluation demonstrated mild leukocytosis with neutrophil predominance. Inflammatory markers were elevated, including erythrocyte sedimentation rate (ESR) and C-reactive protein (CRP). Procalcitonin was slightly above the reference range at 0.10 µg/L; however, per the performing laboratory, values ranging from 0.10 to 0.25 µg/L indicated a low likelihood of bacterial infection. Infectious workup revealed an isolated positive mumps IgM antibody; however, testing for EBV (mononucleosis), rapid streptococcal antigen, COVID-19, influenza, gonorrhea, chlamydia, HIV, and hepatitis B and C was negative. Thyroid function testing and pregnancy testing were normal. D-dimer and troponin were negative, which were felt to make a pulmonary embolism or a cardiac ischemic process less likely, respectively (Table 1).

**Table 1:** Relevant serum laboratory test values during urgent care presentation

Laboratory test	Patient Value	Reference Range
White blood cell (WBC) count	10.35 x 10^3^/µL	4.16-9.95 x 10^3^/µL
Absolute neutrophil count	8.02 x 10^3^/µL	1.80-6.90 x 10^3^/µL
Hemoglobin	12.2 g/dL	11.6-15.2 g/dL
Platelet count	285 x 10^3^/µL	143-398 x 10^3^/µL
Erythrocyte sedimentation rate (ESR)	64 mm/hr	≤25 mm/hr
C-reactive protein (CRP)	6.5 mg/dL	<0.8 mg/dL
Procalcitonin	0.10 µg/L	<0.10 µg/L
Mumps virus antibody (IgM)	1.42 IV	≤0.79 IV
Thyroid-stimulating hormone (TSH)	2.9 mcIU/mL	0.3-4.7 mcIU/mL
D-dimer	0.49 µg/mL FEU	<0.60 µg/mL FEU
High sensitivity troponin I	<4 ng/L	<5 ng/L

A chest radiograph was obtained on the day of presentation and showed no acute cardiopulmonary processes, such as pneumothorax, pulmonary lesions, or pleural effusion. Subsequently, a same-day computed tomography (CT) of the neck with contrast demonstrated multistation cervical lymphadenopathy involving right level IV and left levels II-V lymph node stations, with the largest node at left level IV measuring approximately 14 × 19 mm. CT imaging also revealed multiple enlarged left axillary lymph nodes, the largest measuring approximately 20 × 30 mm, as well as a prominent lymph node conglomerate in the partially visualized mediastinum. These findings were felt to be concerning for a lymphoproliferative process according to the radiologist report (Figure 1 and Figure 2).

**Figure 1:**
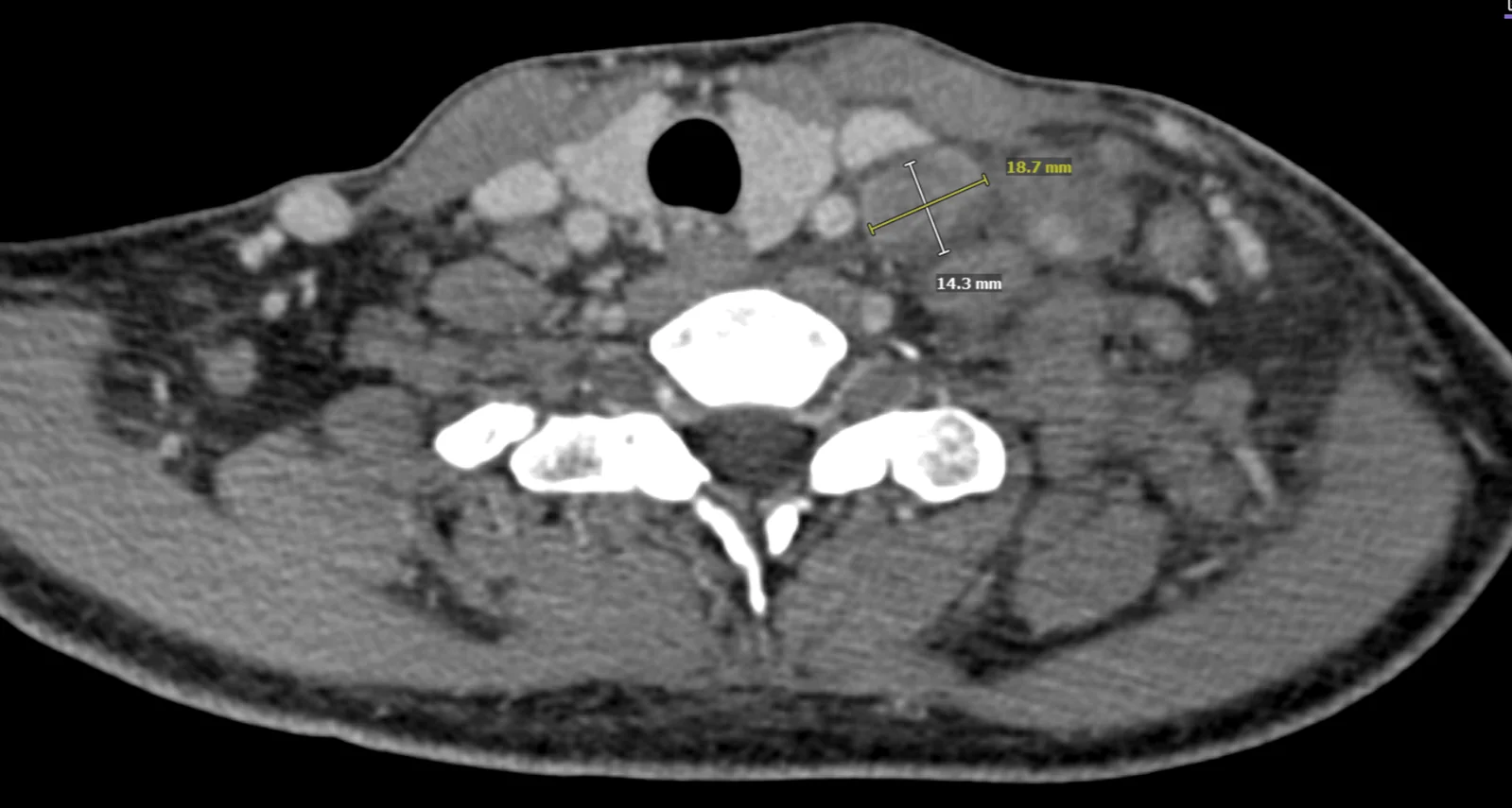
Key image from CT neck with contrast Left level IV neck lymphadenopathy with the largest node measuring 14.3 × 18.7 mm in the transverse axis (white and yellow calipers).

**Figure 2:**
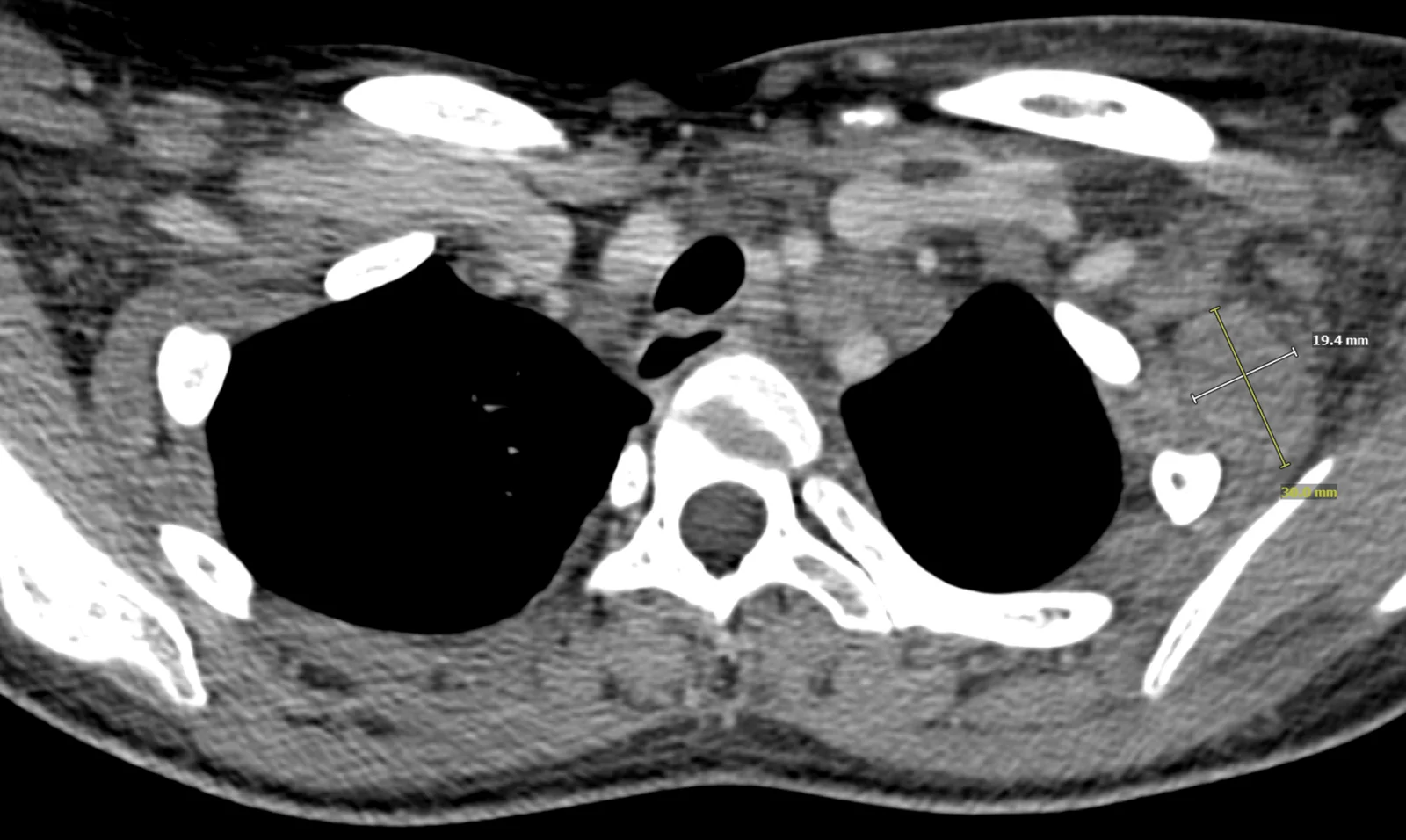
Key image from CT neck with contrast Left axillary lymphadenopathy with the largest node measuring 19.4 × 30.0 mm in the transverse axis (white and yellow calipers).

In light of these findings, the patient was urgently referred to an oncology specialist the next day, who recommended an axillary lymph node biopsy to definitively rule out a lymphoproliferative process such as lymphoma. One day after the initial presentation, a diagnostic ultrasound with biopsy of the left axilla was then performed, which demonstrated numerous enlarged lymph nodes corresponding to the patient’s palpable left axillary masses with cortical thickening and diminished fatty hila, with the largest measuring approximately 29 × 14 × 25 mm and demonstrating internal vascularity (Figure 3).

**Figure 3:**
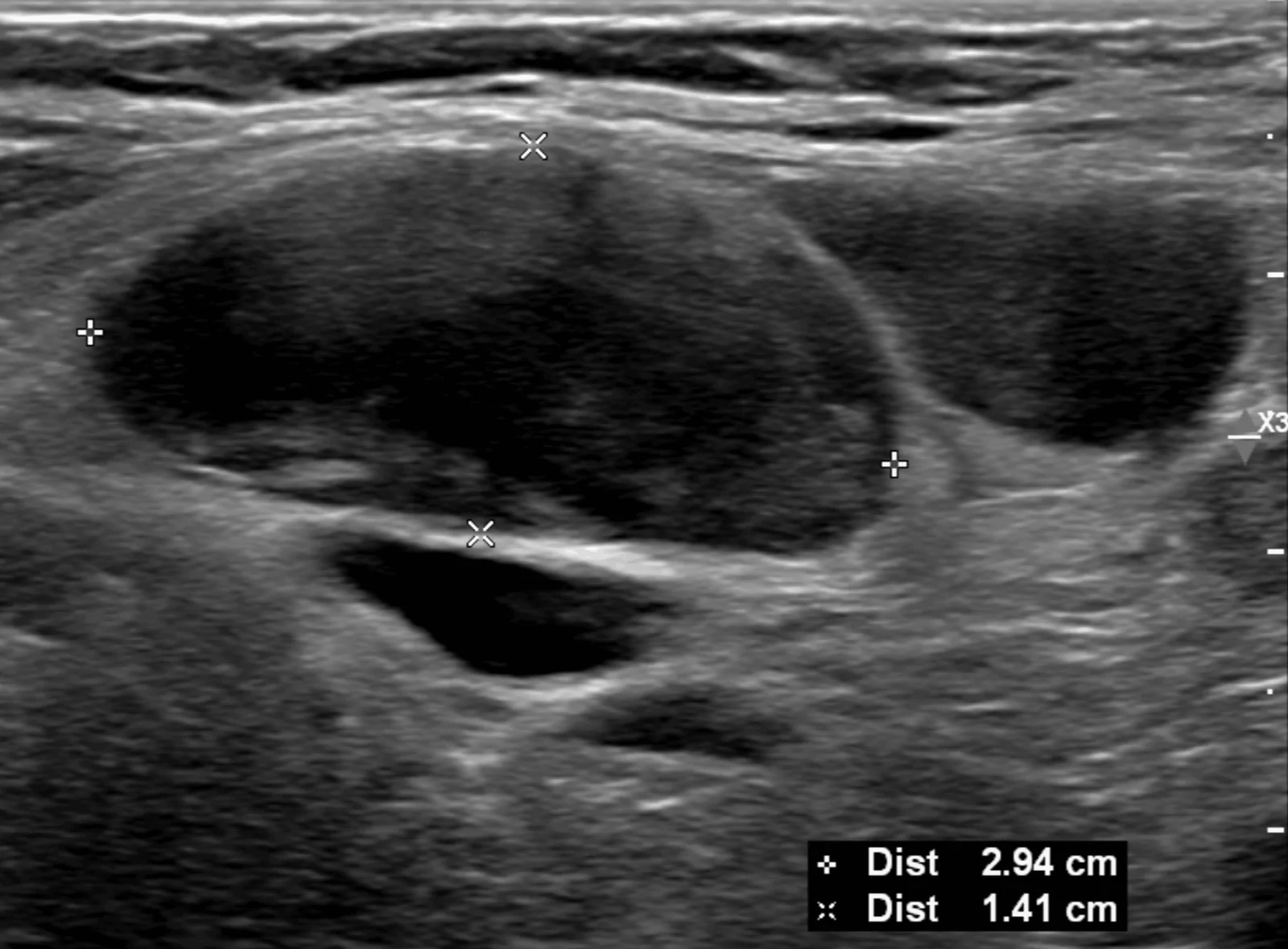
Key image from ultrasound left breast Lymphadenopathy with the largest lymph node measuring 29.4 × 14.1 mm (+ and x calipers).

A core needle biopsy of a left axillary lymph node was obtained at the time of the diagnostic ultrasound, which revealed focal lymphoid tissue with numerous eosinophils, with no diagnostic evidence of lymphoma; however, the specimen showed limited lymph node sampling. Given that this result was considered discordant with the patient’s clinical presentation, the risks and benefits of repeat core needle biopsy versus excisional biopsy were discussed with the patient, who preferred repeat core needle biopsy. Although repeat biopsy of the left axillary lymph node was considered, the left supraclavicular lymph nodes were felt to be more easily accessible under ultrasound guidance and were thought to likely represent the same underlying process. Therefore, an ultrasound-guided core needle biopsy of a left level IV supraclavicular lymph node was subsequently performed nine days after the initial presentation (Figure 4).

**Figure 4:**
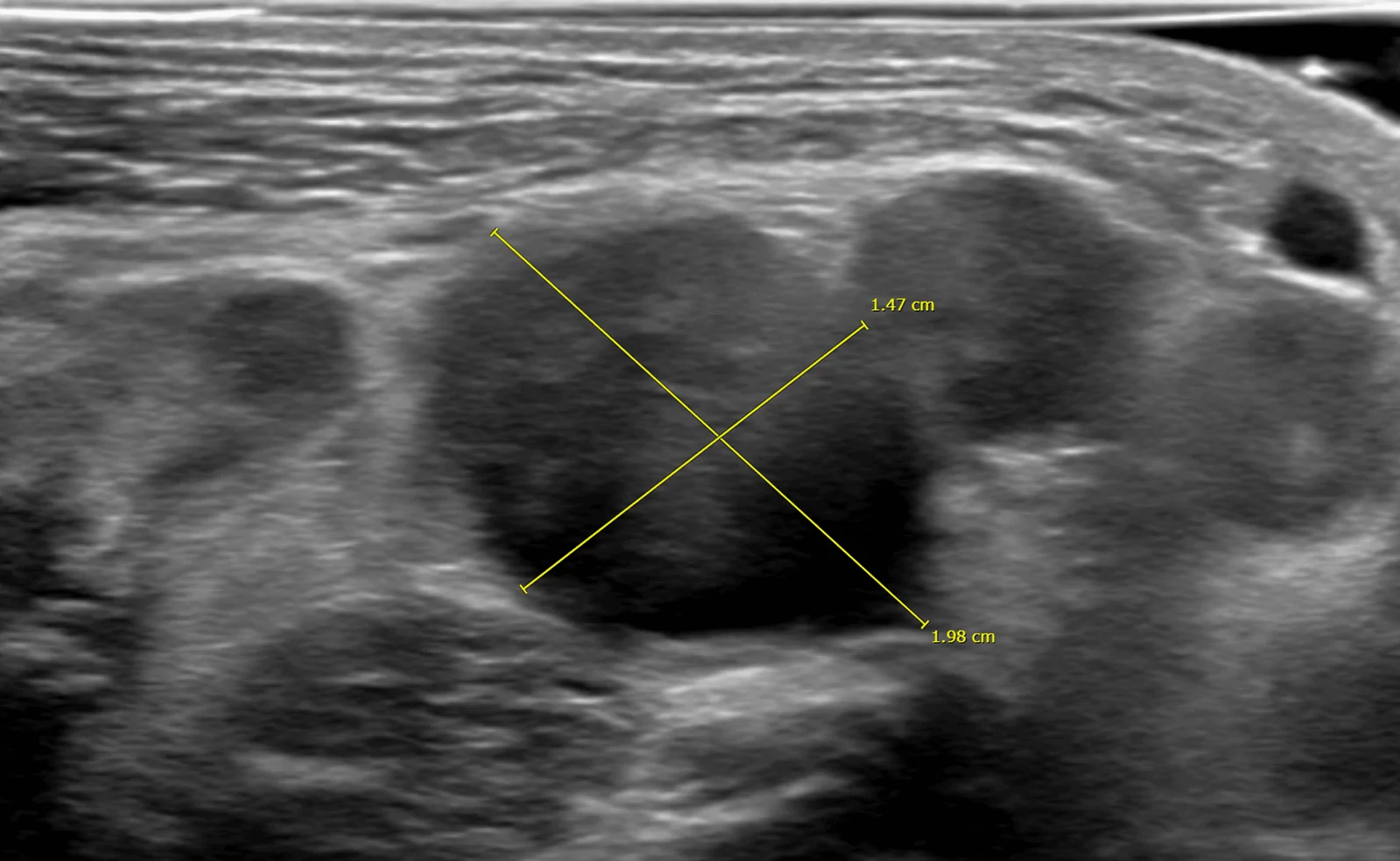
Key image from ultrasound left neck Lymphadenopathy with the largest lymph node measuring 14.7 × 19.8 mm (yellow calipers).

Histopathologic evaluation demonstrated dense sclerosis with a nodular architecture and scattered foci containing cells with abnormal nuclear lobation, irregular nuclear membranes, and prominent eosinophilic nucleoli. Immunohistochemical stains highlighted neoplastic cells that were positive for CD15, CD30, PAX5, BOB1, and MUM1, confirming findings consistent with classical Hodgkin lymphoma, nodular sclerosis subtype, and were negative for EBV by in situ hybridization. Concurrent flow cytometric studies also revealed no monotypic B-cell population and no pan T-cell aberrancy.

Fifteen days after initial presentation, positron emission tomography (PET)-CT scan demonstrated numerous multistation lymphadenopathies with variable moderate to intense fluorodeoxyglucose (FDG) uptake involving the bilateral cervical and bilateral axillary regions (left greater than right), as well as the mediastinum, consistent with Hodgkin’s lymphoma (Figure 5).

**Figure 5:**
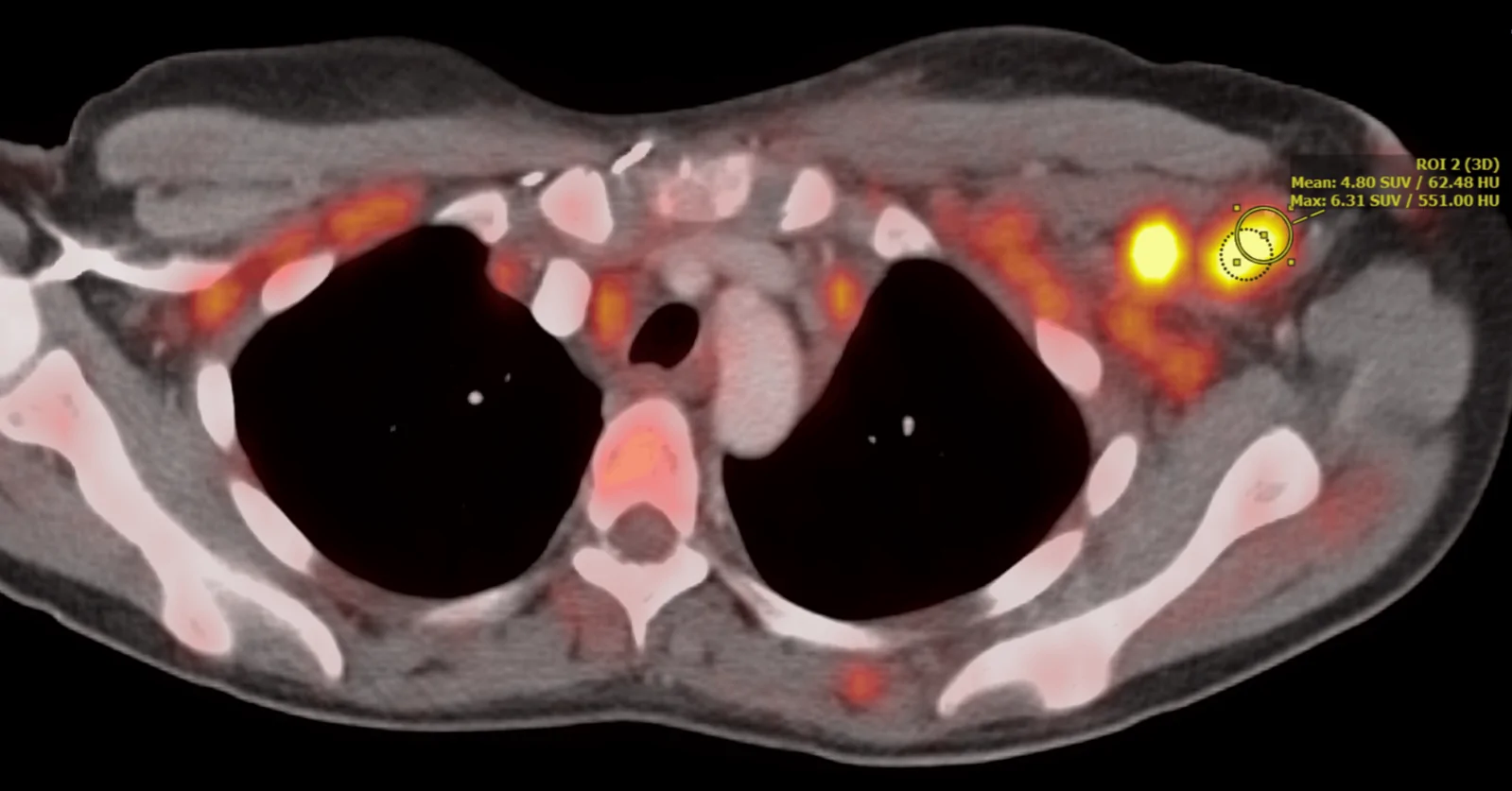
Key image from PET-CT Lymphadenopathy is demonstrated with avid FDG uptake, prominent in the left axillary region (circled). ROI: region of interest; SUV: standardized uptake value; HU: Hounsfield unit; FDG: fluorodeoxyglucose

The patient was subsequently referred to a tertiary care center for further evaluation and treatment and was diagnosed with stage IIA nodular sclerosis classical Hodgkin lymphoma.

## Discussion

Although infectious causes are the most common etiology of lymphadenopathy, certain clinical and radiologic features, including multistation and mediastinal lymphadenopathy, should raise concern for malignancy. In this case, our patient presented with a two-week history of enlarging multistation cervical and axillary lymphadenopathy without B symptoms. Although an isolated positive mumps IgM was identified during the initial evaluation, the overall clinical and radiologic presentation remained concerning for malignancy. Mumps characteristically causes parotitis rather than discrete lymphadenopathy, axillary lymph node involvement is not a recognized feature of mumps, and the patient’s multistation and mediastinal lymphadenopathy was atypical for mumps infection [7]. Further imaging and repeat biopsy ultimately led to the diagnosis of classical Hodgkin lymphoma. This case highlights the diagnostic challenge of lymphadenopathy in urgent care and primary care settings, where infection is common, but malignancy must remain an important consideration when clinical and radiologic findings are concerning for a lymphoproliferative process.

Beyond the clinical discordance, the positive mumps IgM itself warrants careful interpretation. There is no established causal association between mumps virus and Hodgkin lymphoma; rather, the virus mostly implicated is EBV, which is found in 25-40% of classical Hodgkin lymphoma cases [2]. False-positive mumps IgM results can arise from cross-reactivity with other paramyxoviruses, such as parainfluenza viruses, which share antigenic epitopes with the mumps virus [7,8]. In this case, mumps infection was not confirmed by polymerase chain reaction (PCR), repeat serology, IgG testing, or public health testing, which limits the consideration of this diagnosis. In addition, this patient received her measles, mumps, rubella (MMR) vaccine in 2015, and previously immunized individuals who encounter mumps are less likely to mount a robust IgM response, as IgM is not a major component of the secondary immune response [9,10]. Therefore, the isolated positive mumps IgM may have represented a false-positive result rather than evidence of a true acute infection.

The differential diagnosis of lymphadenopathy includes infectious, malignant, autoimmune/inflammatory, iatrogenic, and miscellaneous causes, with infectious etiologies being the most common [4,11]. However, certain clinical features that should raise concern for malignancy include supraclavicular lymphadenopathy, lymph nodes larger than 2 cm, firm or fixed nodes, constitutional symptoms, or multistation lymphadenopathy. In our patient, multistation lymphadenopathy and mediastinal involvement were concerning features that warranted further evaluation.

A systematic approach is essential in the evaluation of lymphadenopathy and should include a thorough history, physical examination, and risk stratification to guide imaging and biopsy decisions. Important historical features include duration of lymphadenopathy, associated symptoms such as B symptoms, hemoptysis, fatigue, rash, and arthralgias, and relevant exposures including travel, animal contact, medications, vaccinations, and sexual history. Physical examination should assess the location of lymphadenopathy (localized vs. generalized), size, texture, mobility, and tenderness, as well as evaluate lymphatic drainage regions, spleen, and liver. High-risk patients should undergo prompt imaging, typically with contrast-enhanced CT in adults, followed by tissue biopsy if imaging findings are suspicious [4]. In this case, imaging findings were concerning for a lymphoproliferative disorder; however, the initial core needle biopsy was nondiagnostic. This discordance between imaging and pathology prompted repeat biopsy, which ultimately established the diagnosis. Importantly, core needle biopsy has diagnostic limitations compared with excisional biopsy in suspected Hodgkin lymphoma, as limited tissue sampling may miss sparse Reed-Sternberg cells and inadequately assess nodal architecture [12]. This case therefore demonstrates that discordant clinical, imaging, and biopsy findings should prompt repeat biopsy when suspicion for malignancy remains high.

Classical Hodgkin lymphoma most commonly manifests as painless cervical or supraclavicular lymphadenopathy and frequently involves the mediastinum [3]. The disease typically spreads in a contiguous pattern between lymph node groups and may present with B symptoms in approximately 25-40% of cases; however, the absence of B symptoms does not exclude the diagnosis [3,13,14]. In this case, the patient had multistation lymphadenopathy involving the cervical, axillary, and mediastinal regions, consistent with the typical distribution pattern of Hodgkin lymphoma. This case is notable because the patient initially presented without B symptoms and had an isolated positive mumps IgM result, each of which could have confounded the initial clinical picture. Additionally, the initial core needle biopsy was limited, highlighting that discordant biopsy results should prompt consideration of repeat biopsy when clinical suspicion remains high. Early recognition and diagnosis are important because Hodgkin lymphoma is highly treatable, with overall survival exceeding 90% in early-stage disease and 75-90% in advanced-stage disease [3].

## Conclusions

This case highlights the importance of maintaining a broad differential diagnosis when evaluating lymphadenopathy, particularly in the presence of concerning features such as large lymph node size, multistation involvement, and mediastinal lymphadenopathy. Although infectious causes are common, isolated positive serologic findings should be interpreted in the context of the overall clinical presentation and should not preclude further evaluation when other findings raise concern for malignancy. This case also demonstrates that the absence of B symptoms does not exclude Hodgkin lymphoma and underscores the importance of recognizing discordance between concerning clinical and imaging findings and an initially limited or nondiagnostic biopsy. When suspicion for malignancy remains high, further evaluation and repeat biopsy should be pursued. Early recognition and accurate diagnosis are critical, as Hodgkin lymphoma is a highly treatable malignancy.
